# Strategies for cystic fibrosis transmembrane conductance regulator inhibition: from molecular mechanisms to treatment for secretory diarrhoeas

**DOI:** 10.1002/1873-3468.13971

**Published:** 2020-11-16

**Authors:** Hugo R. de Jonge, Maria C. Ardelean, Marcel J. C. Bijvelds, Paola Vergani

**Affiliations:** ^1^ Department of Gastroenterology & Hepatology Erasmus University Medical Center Rotterdam The Netherlands; ^2^ Department of Neuroscience, Physiology and Pharmacology University College London UK; ^3^ Department of Natural Sciences University College London UK

**Keywords:** CFTR pharmacology, cholera, cyclic AMP, cyclic GMP, enterocyte, G907 compound, glibenclamide, ion‐channel gating, secretory diarrhea, zosuquidar

## Abstract

Cystic fibrosis transmembrane conductance regulator (CFTR) is an unusual ABC transporter. It acts as an anion‐selective channel that drives osmotic fluid transport across many epithelia. In the gut, CFTR is crucial for maintaining fluid and acid‐base homeostasis, and its activity is tightly controlled by multiple neuro‐endocrine factors. However, microbial toxins can disrupt this intricate control mechanism and trigger protracted activation of CFTR. This results in the massive faecal water loss, metabolic acidosis and dehydration that characterize secretory diarrhoeas, a major cause of malnutrition and death of children under 5 years of age. Compounds that inhibit CFTR could improve emergency treatment of diarrhoeal disease. Drawing on recent structural and functional insight, we discuss how existing CFTR inhibitors function at the molecular and cellular level. We compare their mechanisms of action to those of inhibitors of related ABC transporters, revealing some unexpected features of drug action on CFTR. Although challenges remain, especially relating to the practical effectiveness of currently available CFTR inhibitors, we discuss how recent technological advances might help develop therapies to better address this important global health need.

## Graphical abstract

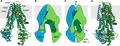

## Abbreviations

**AC**, adenylyl cyclase

**CF**, cystic fibrosis

**CFTR**, cystic fibrosis transmembrane conductance regulator

**CH**, coupling helix

**CT**, cholera toxin

**ETEC**, enterotoxigenic strains of *Escherichia coli*

**GC‐C**, guanylyl cyclase C

**IF**, inward facing

**LT**, heat‐labile enterotoxin

**NBD**, nucleotide binding domain

**NHE3**, Na^+^/H^+^ exchanger 3

**NKCC1**, Na^+^‐K^+^‐Cl^−^ cotransporter 1

**OF**, outward facing

**ORS**, oral rehydration solution

**PDE3**, phosphodiesterase 3

**PKA**, cAMP‐dependent protein kinase

**PKG2**, cGMP‐dependent protein kinase 2

**SD**, secretory diarrhoea

**STa**, heat‐stable enterotoxin

**TM**, transmembrane helix

**TMD**, transmembrane domain

CFTR (cystic fibrosis transmembrane conductance regulator, or ABC‐C7), an unusual ABC transporter that functions as an anion channel [1], controls fluid movement across epithelia [2]. Loss‐of‐function mutations in CFTR cause the dehydrated secretions characteristic of the genetic disease cystic fibrosis (CF) [3]. In contrast, during life‐threatening enterotoxin‐mediated secretory diarrhoeas, over‐activation of CFTR causes excessive fluid secretion across intestinal epithelia [4].

Diarrhoeal diseases are the second most‐common cause of death in young children worldwide. Oral rehydration therapy is very effective. However, where extreme poverty, natural disaster and/or war impede provision of safe water, fatality rate increases dramatically (http://www.who.int/en/news‐room/fact‐sheets/detail/diarrhoeal‐disease). CFTR inhibitors have been shown in model systems to rapidly block fluid loss during enterotoxin‐mediated diarrhoeas (e.g., cholera [5]), and could be useful in preventing fatal dehydration and further disease spread. Here we consider whether they could help in the Global Action Plan towards eliminating childhood preventable deaths (https://www.who.int/maternal_child_adolescent/news_events/news/2013/gappd_launch/en/) when used alongside existing preventive interventions and therapies.

Excellent reviews cover the development, pharmacodynamic and pharmacokinetic aspects of existing CFTR inhibitors [6, 7, 8, 9]. This paper does not aim at being comprehensive. Rather, we focus on mechanism of inhibition, by selecting a small number of compounds, targeting both CFTR and related ABC transporters, which have been investigated in depth. Comparisons of inhibitor mechanisms can shed light on both CFTR and pump protein dynamics, and could inform efforts aimed at developing improved therapies.

## CFTR is a unique ABC system

CFTR is, so far, the only ABC transporter known to function as a channel: it can form an aqueous ion conduction pathway that permits the flow of anions across the plasma membrane down their electrochemical gradient [10]. Structurally, CFTR nevertheless shares highly conserved domains and an overall fold with many transporter relatives [1].

### Domain structure of CFTR

The CFTR coding sequence includes two homologous halves, each formed by a transmembrane domain (TMD) followed by a highly conserved cytosolic nucleotide binding domain (NBD). CFTR has a typical type IV core fold [11], with each TMD comprising 6 transmembrane helices (TMs). But in CFTR the two halves are linked by a ~ 200 amino acid‐long domain with no homology to other proteins, the R‐domain. Some evidence suggests that the emergence of CFTR's channel function coincided with the acquisition of the R‐domain [12]. Indeed, the R domain plays an important role in controlling ion‐channel function: CFTR becomes active only following phosphorylation of specific R domain serine residues, mainly by cAMP‐dependent (and cGMP in the intestine, see CFTR in intestinal epithelia) protein kinases.

As in other ABC systems, binding of ATP at the NBDs favours the formation of a ‘head‐to‐tail’ NBD1/NBD2 dimer, with two nucleotide‐binding sites at the interface. The covalently‐linked γ‐phosphate forms molecular contacts with both NBDs, stabilizing the dimer [13]. Formation of the NBD dimer is associated with conformational changes in the TMDs that result in opening of the ion conduction pathway, as detailed below. An active site, capable of catalysing hydrolysis of the β–γ phosphate bond, is also formed in this dimerized state thus triggering NBD dimer dissociation to complete the conformational cycle [14]. CFTR belongs to a large group of asymmetric ABC transporters, which have only one catalytically active nucleotide‐binding site [15], including canonical, conserved sequence motifs at the NBD1/NBD2 interface (canonical site 2). The other interfacial site (noncanonical, or degenerate site 1) binds ATP tightly, but has lost hydrolytic activity, due to a number of nonconservative substitutions in the sequence motifs [1].

### Gates, channels and pumps

In both pumps and channels, conformational changes result in the movement of specific gate structures. When closed, these block substrate/ion access to the translocation/permeation pathway. For pumps, which couple the energy releasing process of ATP hydrolysis to the thermodynamically uphill movement of a substrate against an electrochemical gradient, it is crucial that none of the conformations visited physiologically form a continuous cytosol‐to‐extracellular pathway, a pore that would allow dissipation of the generated gradient [16].

Indeed, experimentally observed ABC transporter conformations (obtained using X‐ray crystallography and electron cryo‐microscopy, cryo‐EM) mainly belong to two classes, each one presenting at least one closed gate [11, 17]. Inward facing (IF) conformations, in which the NBDs are separated and the TMDs converge at the extracellular side of the membrane, have mostly been obtained in the absence of nucleotides. These reveal a closed outer gate, as the translocation/permeation pathway is continuous with the cytosolic solution but not with the extracellular space. By contrast, in structures largely obtained in the presence of ATP and under conditions that prevent hydrolysis, the cytosolic TMD extensions have been drawn together by NBD dimerization. These are outward‐facing (OF) conformations where the TMDs form tight helical bundles on the cytosolic side of the translocation/permeation pathway. In OF conformations, the inner gate is shut, closing access from the cytosol. In some structures, both inner and outer gates are closed, forming an ‘occluded’ substrate‐binding site closed off to both sides of the membrane (e.g. [18, 19]). For simplicity, herein we classify conformations characterized by dimerized NBDs as belonging to the OF group. Overall, this evidence supports the ‘alternating access’ model of transporter function, with hydrolytic cycles at the NBDs driving coupled conformational changes in the TMDs that gate the translocation pathway in an alternating manner and result in unidirectional transport of substrate across the membrane.

The CFTR channel shares some of the molecular mechanisms used by its pump relatives. However, while in pumps conformational changes are stoichiometrically coupled to the movement of one or a few substrate molecules, in CFTR the homologous conformational changes associated with ATP binding and hydrolysis open and close a continuous permeation pathway, directly linking cytosolic and extracellular spaces. In other words, CFTR has only one functional gate. Once this gate is open, millions of anions can flow per second, down their electrochemical gradient, without needing further slow protein movements to get past a second gate.

### Structural snapshots of CFTR

Patch‐clamp studies, in which gate opening and closing can be followed in real time on individual channels, have revealed that in CFTR, gate opening follows ATP binding at site 2 and is coupled to formation of a tight NBD1/NBD2 dimer [20]. The interface tightens around the ATP bound at site 2 early during the opening transition, while movement around site 1 is more delayed [21, 22]. Finally, hydrolysis of the ATP molecule bound at site 2 triggers NBD dimer dissociation and gate closure [23]. Atomic‐resolution structures of full‐length CFTR have largely confirmed these earlier studies linking NBD dynamics to movement of the channel gate, but have also revealed some idiosyncratic characteristics of this unique ABC system.

**Fig. 1:**
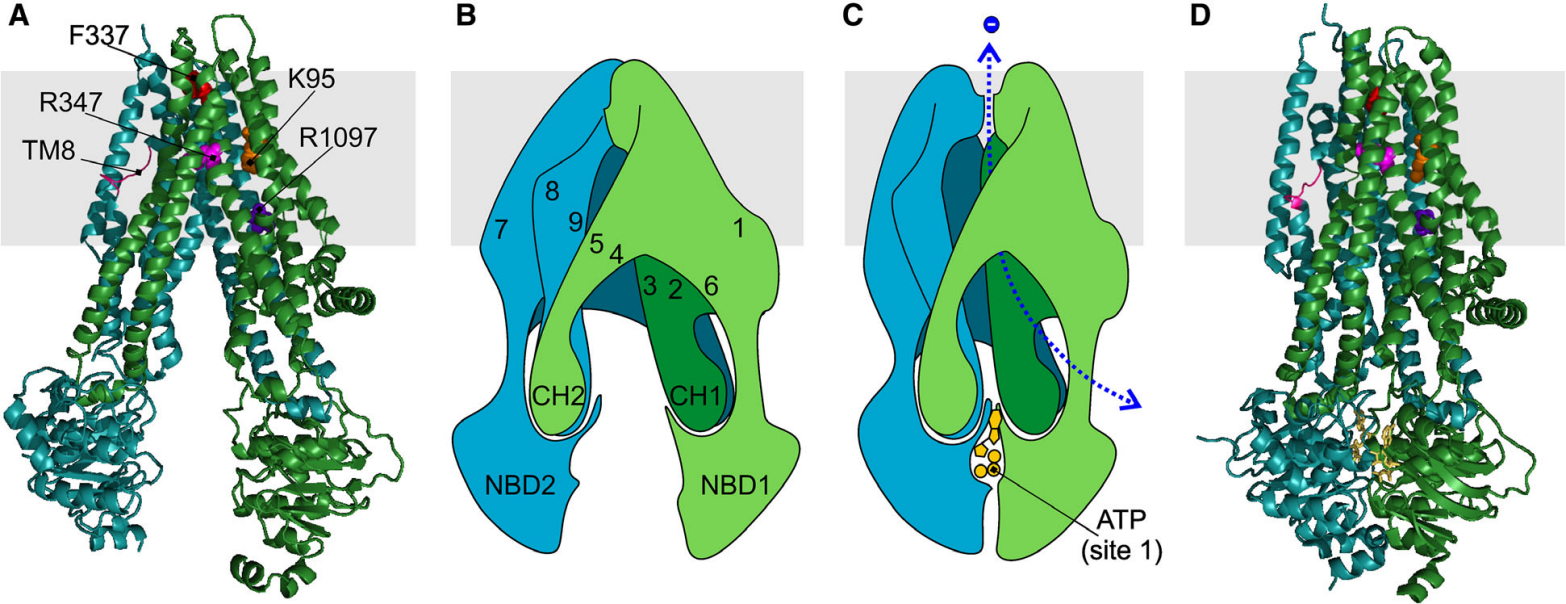
Structural snapshots of CFTR. (A) Cartoon representation of IF CFTR conformation, based on the dephosphorylated, ATP‐free cryo‐EM model of human CFTR (PDB ID5UAK, [27]). TMD1‐NBD1, green; TMD2‐NBD2, teal. Some residues mentioned in text are shown as coloured spheres. Also highlighted is the unwound portion of TM8 (magenta). The density corresponding to the R domain, which in this conformation is located between the two TMDs, at a level below the cytosolic face of the membrane (grey band), is omitted for clarity. (B) Schematic representation of IF conformation shown in A. Numbers indicate positions of transmembrane helices. (C) Schematic representation of OF, open channel CFTR conformation. ATP is shown in yellow. The degenerate site 1 is here on the front, while canonical site 2, also occupied by ATP, is on back (not shown). Here, an open anion permeation pathway is indicated (dotted blue line), based on extensive functional evidence linking NBD dimerization to channel opening. However, we still lack structural evidence of a corresponding OF, open‐pore CFTR conformation, as in PDB ID6MSM, see D, the extracellular end of the permeation pathway is obstructed. (D) Cartoon representation of phosphorylated, ATP‐bound human E1371Q‐CFTR (6MSM, [30]). The R domain density is again not shown. In this view it lies on the back of the protein, at a level below the lasso domain (here visible as the short helix parallel to the plane of the membrane, on right of image).

As in other ABC systems, IF conformations with separated NBDs (Fig. 1A,B) and OF conformations with a tight NBD1/NBD2 dimer (Fig. 1C,D) have been observed. Each TMD includes two short ‘coupling helices’ (CHs) at the far intracellular end of the TMs. These are positioned within depressions in the NBDs to form ‘ball‐and‐socket’ joints. For each TMD, the most C‐terminal CH ‘domain‐swaps’: CH1 + CH4 interface with NBD1, CH2 + CH3 with NBD2 (Fig. 1B). The residues involved in anion permeation delineate a clear permeation pathway [24], consistent with results from decades of functional experiments (reviewed in Refs [25, 26]). As expected, dephosphorylated, ATP‐free conformations show separated NBDs and a permeation pathway closed off on the extracellular side [24, 27]. The extracellular CFTR gate is closed [28, 29]. In these IF conformations, the R domain is detected as a largely unstructured electron‐dense region which occupies space in between the two structural halves (TMs 1, 2, 3, 6, 10, 11 with NBD1 and TMs 7, 8, 9, 12, 4, 5 with NBD2, Fig. 1B). Effectively, the dephosphorylated R domain sterically hinders the IF‐to‐OF transition driven by formation of an NBD1/NBD2 dimer and therefore prevents the opening of the gate.

R domain phosphorylation causes a shift in the R domain density [30, 31]. In the phosphorylated structures (obtained in the presence of ATP and carrying the E1371Q mutation that prevents hydrolysis), as observed for the OF conformations of other Type IV ABC systems, the two structural halves have rotated towards each other and a typical head‐to‐tail NBD dimer is formed (Fig. 1C). Atypically, however, in CFTR NBD‐dimerized, OF structures, the inner gate is not closed: access to the permeation pathway from the cytosol is provided by a bypass ‘portal’ that opens between TM4 and TM6 [32, 33].

Another unusual feature, present in both IF and OF CFTR structures, is an unwound segment in TM8. This displaces TM7, and forms a groove on the membrane‐facing surface of the TMDs. Even CFTR's close pump relative, multidrug resistance‐associated protein 1 (MRP1 or ABC‐C1), does not share this feature [27]. Microsecond‐long molecular dynamics simulations with the protein embedded in a lipid bilayer suggest the unwinding of TM8 is relatively stable [34].

Finally, OF CFTR structures were found to have a closed extracellular gate. This was unexpected, as a large body of experimental evidence suggests that the conditions in which the structures were obtained (following phosphorylation, in the presence of Mg^2+^ATP, with Walker B catalytic site 2 ‘E‐to‐Q’ mutation E1371Q) strongly stabilize a conformation with an open permeation pathway [20]. It is possible that the absence of a proper lipid bilayer during preparation of the cryo‐EM sample has altered the relative stability of alternative conformations, and the OF cryo‐EM structures reflect the conformation adopted in the physiologically short‐lived intraburst closures [31], or a relatively stable ‘pre‐open’ conformation that contributes to the final stretch of the long interburst closed dwell‐times observed in single‐channel records [35]. In any case, we do not have a complete molecular understanding of CFTR's open outer gate.

## CFTR in intestinal epithelia

### Transport processes in enterocytes

A vitally important function of mammalian intestinal epithelia is the isotonic secretion of fluid and electrolytes, consisting mainly of Na^+^, Cl^−^ and ${\text{HCO}}_{3}^{-}$ ions. This process not only promotes the enzymatic digestion of nutrients and prevents dehydration of the epithelial surface and intestinal obstruction but, in concert with the propulsive movements of the intestinal smooth muscle, also serves to protect the intestinal tract against noxious agents, including bacteria and their enterotoxins.

**Fig. 2:**
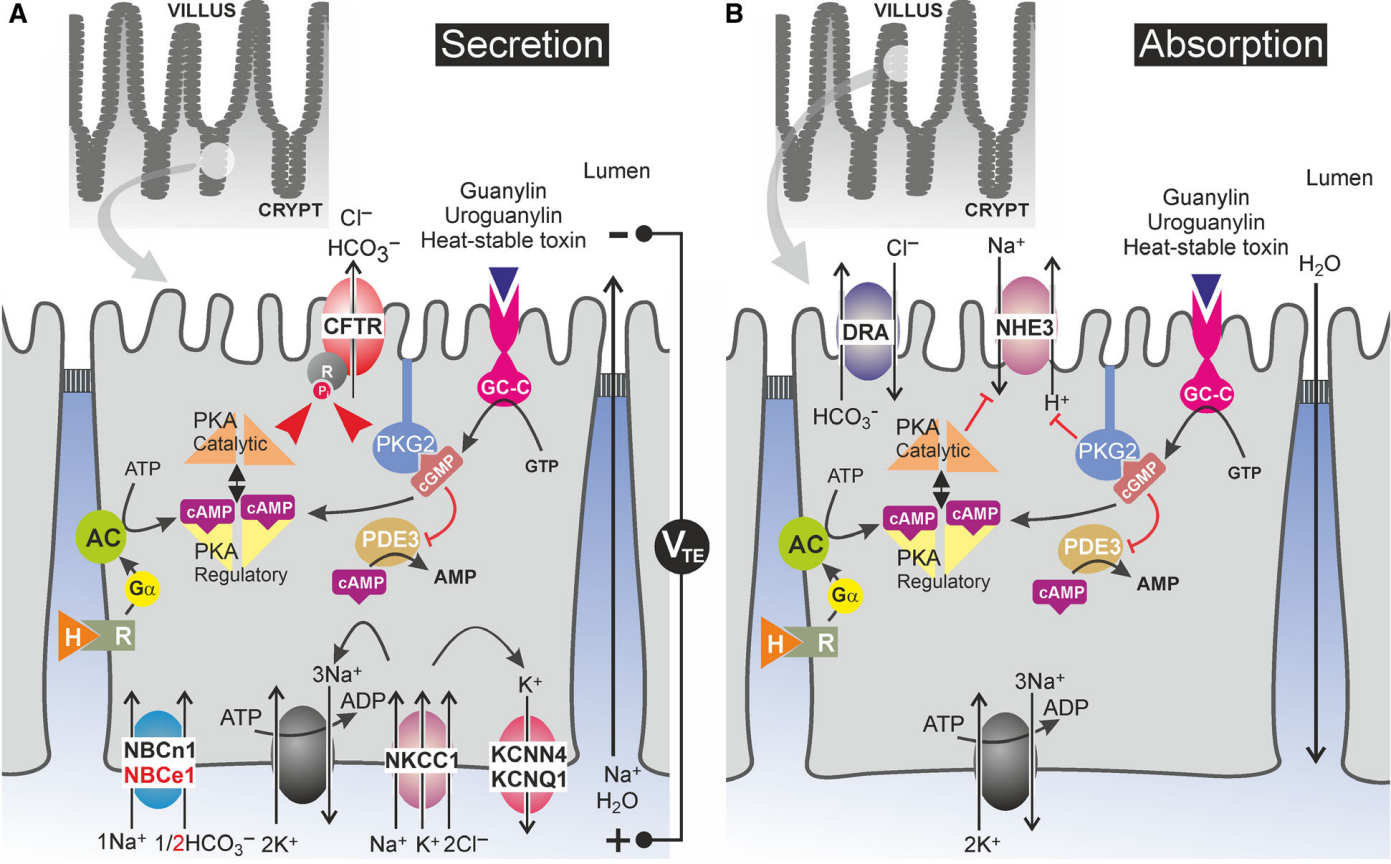
Location in enterocytes of ion transporters and cyclic nucleotide signalling cascades involved in enterotoxin‐induced intestinal electrolyte and fluid secretion. (A) CFTR‐mediated vectorial anion secretion in CFTR‐enriched crypt cells. The Na^+^,K^+^‐ATPase provides the driving force for basolateral Cl^−^and${\text{HCO}}_{3}^{-}$entry*via*Na^+^‐coupled cotransport, mediated by NKCC1 and NBCe1/NBCn1, respectively. Cl^−^and${\text{HCO}}_{3}^{-}$exit the cell*via*CFTR. In addition,${\text{HCO}}_{3}^{-}$also exits the cell*via* ${\text{Cl}}^{-}/{\text{HCO}}_{3}^{-}$exchangers (DRA, PAT‐1; not shown). Transcellular, electrogenic anion secretion generates a lumen‐negative transepithelial potential difference (V_TE_) that drives passive paracellular Na^+^secretion. The resulting osmotic gradient drives water movement across the tight junctions. Salt and water secretion are regulated by a plethora of neuro‐endocrine factors that control protein kinase‐mediated phosphorylation/activation of CFTR. (B) NHE3‐ and DRA‐mediated NaCl absorption in villus cells. In villus cells, the coordinated activity of NHE3 and${\text{Cl}}^{-}/{\text{HCO}}_{3}^{-}$exchangers (SLC26A3/DRA; and SLC26A6/PAT‐1, not shown) mediates vectorial NaCl uptake, which, in turn, promotes water absorption. cAMP‐ and cGMP‐linked signal transduction routes, that is the same pathways that activate CFTR (also present in the upper epithelium, but at comparatively low levels; see Fig. 3A), inhibit NHE3 and promote${\text{HCO}}_{3}^{-}$secretion. The regulation of${\text{Cl}}^{-}/{\text{HCO}}_{3}^{-}$exchangers is less well defined. AC, adenylyl cyclase; GC‐C, guanylyl cyclase C; H, hormone or para‐/neurocrine factor; PKA, cAMP‐dependent protein kinase, PKG2, cGMP‐dependent protein kinase 2; PDE3, phosphodiesterase 3.

The secretory function is confined mainly to the crypt region and is anatomically separated from the villus compartment whose major transport function is the Na^+^‐ or H^+^‐coupled absorption of digestive products and the reabsorption of water and electrolytes (see model Fig. 2 and [36]). Like in most other Cl^−^ secreting epithelia, the Na^+^, K^+^ ATPase in the basolateral membrane of the crypt cells provides the driving force for Cl^−^ entry *via* the electroneutral cotransporter NKCC1 (Fig. 2A). K^+^ recycles back through basolateral K^+^ channels (KCNQ1; KCNN4), and Cl^−^ exits the cell *via* the CFTR Cl^−^ channel in the apical membrane, a process that can be measured *in vitro* in Ussing chambers as a change in the electrical potential difference across the epithelium or, in voltage‐clamp mode, as a short‐circuit current (*I*_sc_). Similarly, ${\text{HCO}}_{3}^{-}$ can enter the cell *via* Na^+^‐coupled electrogenic (NBCe1) or electroneutral (NBCn1) cotransporters and exit the cell *via* CFTR or ${\text{Cl}}^{-}/{\text{HCO}}_{3}^{-}$ exchangers of the SLC26 family (SLC26A3/DRA; SLC26A6/PAT‐1). Passive Na^+^ secretion occurs paracellularly through cation‐selective tight junctions between the cells, in response to the lumen‐negative transepithelial potential difference resulting from active, transcellular anion secretion. The resulting osmotic gradient drives passive water movement across the ‘leaky’ tight junctions (Fig. 2A).

Under physiological conditions, intestinal electrolyte and fluid secretion from the crypts is in balance with reabsorption in the villus (Fig. 2B). However, in a pathological condition named secretory diarrhoea (SD) secretion is hyperstimulated and absorption inhibited in response to endogenous or exogenous secretagogues, including microbial toxins such as cholera toxin and *Escherichia coli* enterotoxins (see [36] for a complete list). In human SD, fluid loss can exceed 1 L·h^−1^, resulting in rapid systemic dehydration, metabolic acidosis, hypokalemia, and cardiac and renal failure [4].

The key player in most forms of human SD is the CFTR anion channel. In CF, loss‐of‐function mutations in CFTR cause a severe impairment of Cl^−^ and ${\text{HCO}}_{3}^{-}$ secretion in virtually all epithelial tissues and the complete loss of electrolyte and water secretion in intestinal epithelium in which compensatory apical anion channels are lacking ([37, 38]; Fig. 2). The high propensity of CF patients to develop intestinal blockade, manifesting as meconium ileus in newborns and distal intestinal obstruction syndrome in adults, illustrates the key role of CFTR in intestinal electrolyte and fluid homeostasis and identifies this channel, or components of its activation mechanism, as suitable targets for antidiarrhoeal drug therapy [39, 40].

### Localization and function of CFTR

**Fig. 3:**
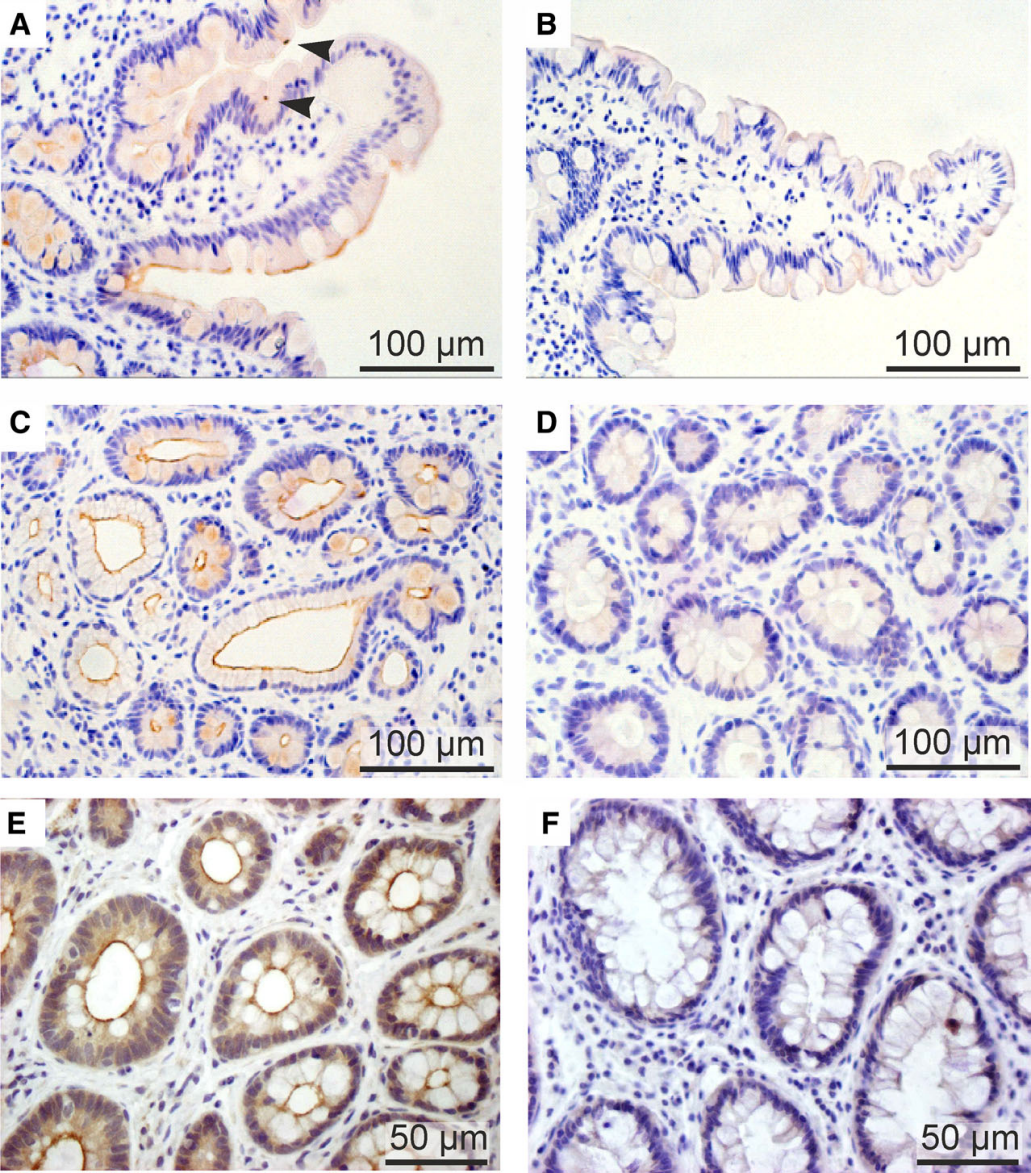
Immunodetection of CFTR in human small intestine and colon. Jejunal (panels A–D) and rectal (panels E and F) biopsies were fixed in performic acid, paraffine embedded and stained with the polyclonal, affinity‐purified hCFTR antibody CC24 [165]. A rabbit specific horseradish peroxidase (HRP)/3,3′‐diaminobenzidine (DAB) detection IHC Kit (Abcam, Cambridge, UK) was used to visualize CFTR protein (brown stain). (A) CFTR expression in jejunal villi showing (a) the absence of CFTR staining in the apical border of goblet cells, and (b) detection of relatively rare CFTR high expressor (CHE) cells (arrow heads). (C) High expression of CFTR protein in the luminal membrane of jejunal crypt cells. (E) CFTR staining in mid‐crypt cells of distal colon/rectum, showing the absence of CFTR in goblet cells. Panels B, D and F show the absence of CFTR immunostaining in biopsies from a homozygous F508del CF patient, confirming the high specificity of the CFTR antibody.

As illustrated in Fig. 3, the CFTR protein is highly expressed in the apical membrane of crypt epithelial cells, along the whole length of the intestine. Single cell RNASeq and functional studies confirm that CFTR is already expressed highly in LGR5^+^ intestinal stem cells at the bottom of the crypt and remains high in all stem cell‐derived cell lineages with the clear exception of mucin‐secreting goblet cells and absorptive enterocytes ([41, 42]; cf. Fig. 3). Furthermore, in human and rat, but not in mouse proximal small intestine, a very rare cell type termed ‘CFTR high‐expresser’ (CHE) cell, resembling pulmonary ionocytes in the airways [43], has been identified ([44]; Fig. 3A). Its precise function has not been elucidated yet but it could be involved in cAMP‐ and Ca^2+^‐regulated local Cl^−^ and fluid secretion serving to clear adherent mucus from intervillous spaces and to facilitate nutrient absorption in the villus compartment [44].

Whereas CFTR‐mediated Cl^−^ secretion serves to move mucus and Paneth cell‐derived defensins out of the intestinal crypts, studies in CF animal models revealed that CFTR‐dependent ${\text{HCO}}_{3}^{-}$ secretion is required for the unfolding and release of mucins from neighbouring goblet cells (stained negatively for CFTR; Fig. 3A,E) and to prevent the formation of viscous and sticky mucus [45]. In addition, CFTR acts as a tumour suppressor gene by preventing intracellular alkalinization and hyperproliferation of intestinal stem cells [42, 46].

### CFTR regulation by cAMP‐ and cGMP‐dependent protein kinases

Upon multisite phosphorylation of serine residues in the R domain by membrane‐bound isoforms of cAMP‐ and cGMP‐dependent protein kinases (PKA2 and PKG2, respectively), the R domain becomes more disordered and no longer hinders conformational changes needed for channel opening [31]. However, in the intestine, in contrast to most other epithelial tissues, increasing the open probability of the CFTR channel is not the sole mechanism by which cAMP and cGMP signalling stimulate CFTR function. The microtubule‐dependent recruitment of CFTR‐rich endosomal vesicles to the apical membrane, involving molecular motor proteins like myosin 1a, is well documented in intestinal cell lines and native intestine and results in a vast increase in the density of CFTR channels on the cell surface [47, 48].

Whereas cAMP/PKA‐induced phosphorylation and activation of CFTR is rather universal among epithelial tissues, cGMP‐triggered activation is confined mainly to enterocytes that express high amounts of the type 2 isoform of PKG [49, 50]. However, in cells in which PKG2 is low or absent, for example in distal colon or in the shark rectal gland, cGMP still activates CFTR through cross talk to the cAMP signalling pathway, either through cGMP activation of PKA or through cGMP inhibition of cAMP hydrolysis by phosphodiesterase 3, most plausibly PDE3B ([51, 52]; Fig. 2). Whereas cGMP signalling in most tissues is triggered by nitric oxide or atriopeptins, intestinal cGMP formation is brought about by a unique class of cysteine‐rich luminocrinic peptides named guanylins [36]. The two major isoforms, guanylin (*GUCA2A*) and uroguanylin (*GUCA2B*), are produced locally by multiple epithelial cell types along the rostrocaudal and crypt‐villus axes [53]. They act as nonabsorbable fluid volume sensors and assist in maintaining fluid homeostasis in the intestinal lumen by binding to a receptor guanylyl cyclase (GC‐C/*GUCY2C*) which is co‐localized with CFTR in the apical membrane [54, 55]. Binding of guanylins triggers conformational changes in the catalytic domain of GC‐C in the cell interior resulting in local elevation of cGMP in close proximity to the CFTR channel. This cGMP‐linked mode of Cl^−^ secretion differs spatially from cAMP‐induced anion secretion in that most endogenous cAMP‐linked hormones and neurotransmitters, for example VIP or prostaglandins, act through G_S_ protein‐coupled activation of adenylyl cyclase isoforms located in the basolateral membrane and require diffusion of cAMP or the catalytic subunit of PKA2 to the target transporters in the luminal membrane ([36]; cf. Fig. 2).

Importantly, cAMP‐ and cGMP‐linked secretagogues exert a dual action: they not only provoke anion secretion by opening of the CFTR channel but additionally inhibit Na^+^ absorption at the level of the Na^+^/H^+^ exchanger NHE3, thereby contributing to net electrolyte and fluid loss in SD ([56, 57]; cf. Fig. 2). Thus, inhibition of cAMP or cGMP signalling is a more effective, albeit less selective, means of counteracting SD in comparison with direct targeting of CFTR by CFTR inhibitors, because it not only results in inhibition of secretion but additionally promotes the restoration of NaCl and water absorption.

### Involvement of CFTR in the pathophysiology of enterotoxin‐mediated diarrhoeas

Multiple enterotoxins secreted by pathogenic bacterial strains colonizing the intestinal wall exploit the cAMP or the cGMP signalling pathway in the enterocyte to elicit excessive salt and water secretion as a flush‐through mechanism to promote their own dissemination. The classical example is a toxin secreted by pathogenic strains of *Vibrio cholerae*, named cholera toxin (CT), which belongs to the AB_5_ group of enterotoxins [4, 36]. Whereas the B pentamer binds to GM_1_ gangliosides on the cell surface, causing the holotoxin to internalize *via* caveolin‐mediated endocytosis, the A subunit becomes unfolded in the ER, is released by retrograde transport into the cytosol, and catalyses NAD‐dependent ADP‐ribosylation of the stimulatory G protein Gαs. This results in irreversible inactivation of the GTPase site and sustained activation of adenylyl cyclase isoform 6 (AC6) which is physically associated with CFTR in the apical membrane [58]. This leads to a permanent elevation of cAMP, PKA and CFTR activity and inhibition of NHE3 during the life span of the host cell (~5 days for human enterocytes). However, due to the rapid renewal of the intestinal epithelium enabled by daily divisions of the stem cells at the bottom of the crypt, cholera is a self‐limiting disease [4, 36].

Importantly, animal studies with labelled CT have shown that penetration of the toxin into the intestinal crypt, the main site of CFTR expression, is slow and incomplete, but nevertheless the net fluid secretion elicited by CT is abundant. This paradox was resolved by the finding that CT is also able to stimulate enterochromaffin (EC) cells in the villus compartment to release 5‐hydroxytryptamine (5‐HT) which activates VIPergic neurons in the myenteric plexus [59]. Both 5‐HT and VIP are potent secretagogues, acting at least in part *via* the cAMP signalling cascade [36, 59]. In addition, activation of VIPergic neurons may evoke a secreto‐motor reflex in the enteric nervous system (ENS), explaining why local CT instillation in the small intestine can elicit fluid secretion in the colon [60]. In summary, studies in animal models show that up to 60% of the intestinal fluid secretory response to CT can be explained by the stimulation of enteric nerves [36, 59]. Furthermore, the complete lack of CT‐induced secretion in the intestine of Cftr^−/−^ mice illustrates that CFTR plays a central role in both ENS‐dependent and ENS‐independent fluid secretion [61].

Of note, multiple other pathogens release CT‐like AB_5_ enterotoxins and cause cAMP‐mediated SD. The most notable of them, heat‐labile enterotoxin (LT) is produced by enterotoxigenic strains of *E. coli* (ETEC) that cause 280 million cases of SD and 370 000 fatalities per year, most of them young children [36].

Aside from CT, *V. cholerae* secretes many other toxins and virulence factors that do not target CFTR but contribute substantially to the pathogenesis of cholera. They include the accessory cholera toxin (ACE) targeting the TMEM16F/ANO6 Cl^−^ channel *via* a RhoGEF‐RhoA‐Rock‐PIP5 kinase pathway [62], and the zonula occludens toxin (Zot) and 2 other toxins that polymerize or cross‐link actin and destabilize the tight junction component ZO‐1 [36]. Importantly, because none of these toxins acts through cAMP and CFTR, their action is insensitive to CFTR inhibitors.

Another major toxin elaborated by ETEC strains, the heat‐stable enterotoxin STa, is a small, poorly immunogenic peptide of 18–19 amino acids containing six cysteine residues and three disulfide bonds [55, 63]. This toxin mimics the action of endogenously produced guanylins by binding in a reversible fashion to the receptor domain of GC‐C, resulting in cGMP formation [64]. As predicted, mice deficient in GC‐C are STa‐ but not CT‐resistant [65]. In contrast, PKG2 ablation in mice reduced the effect of STa on jejunal *I*_sc_ by 80%, but diminished jejunal fluid loss *in vivo* by only 50%, most likely due to a CT‐like action of STa on 5‐HT release by EC cells, followed by an ENS‐mediated release of VIP that signals through cAMP, not cGMP [66, 67, 68].

Despite the wealth of detailed information about the molecular basis of SD summarized above, current treatment options remain highly limited and new, more effective pharmacological therapies are urgently needed.

### Current and potential therapies for secretory diarrhoea

#### Oral rehydration solution

Vaccination campaigns and improved sanitation may help to prevent or limit the devastating effects of a cholera outbreak, but the current mainstay in treating acute diarrhoeal diseases is the administration of an oral rehydration solution (ORS). This inexpensive, easy to implement remedy helped to reduce SD‐related mortality in children below 5 years of age from 4.6 million in 1980 to current levels of 1.2 million per year worldwide [36, 69]. The WHO‐UNICEF‐recommended ORS formulation is hypotonic and contains an equimolar solution of glucose and Na^+^ salts (NaCl and Na‐citrate). Its efficacy is due to the stimulation of Na^+^‐coupled glucose and fluid absorption *via* the SGLT1 transporter, which is not inhibited by cAMP or cGMP signals in the intestinal villi [36]. Active SGLT1 also promotes the recruitment of NHE3 to the apical membrane and uncouples it from CT/cAMP inhibition *via* an AKT/NHERF2‐dependent mechanism, further stimulating NaCl and fluid absorption [70]. ORS supplementation with Zn^2+^ and amylase‐resistant starch further improves the efficacy of ORS, the latter acting by promoting the release and uptake of short‐chain fatty acids produced from starch by commensal bacteria in the colon [71].

Unfortunately, despite its efficacy in systemic rehydration, ORS does not reduce the duration or volume of diarrhoea and therefore negatively affects compliance to the therapy [72] and increases the risk of further spread of infection. On the other hand, it promotes the removal of the pathogen from the individual and avoids accretion of fluid and dangerous distension of intestinal loops [4]. Clearly, new, safe, effective and affordable drug therapies are needed to complement or replace ORS, particularly to combat traveller's diarrhoea or SD in developing countries. Such therapies should target crucial steps in the mechanism by which microbial enterotoxins cause SD in the host, but avoid adverse or toxic side effects in the intestine or other organs. Before discussing inhibition of CFTR, hyperactivation of which plays a crucial role in all types of human SDs, we present alternative potential targets for therapy.

#### Inhibitors of other ion channels or transporters

One obvious class of potential antisecretory drug targets are the ion channels and transporters depicted in Fig. 2. Aside from CFTR, other ion transporters suitable as drug targets in SD include the apical Na^+^/H^+^ exchanger NHE3, the basolateral Cl^−^ importer NKCC1, and the basolateral K^+^ channels KCNQ1 and KCNN4, which hyperpolarize the cell membrane and enhance the electrical driving force for Cl^−^ exit through the CFTR channel [73]. A peptide that mimics part of the NHE3 C‐terminal domain and acts as a dominant NHE3 agonist is under development and could serve as a proabsorptive therapeutic [74]. NKCC inhibitors such as bumetanide are already used as diuretics in the clinic, counteracting their potential utility in preventing SD‐associated systemic dehydration. However, if it is feasible to develop more selective NKCC1 inhibitors that do not cross‐react with NKCC2, the major NKCC isoform in the kidney, such compounds would offer great promise as novel antidiarrhoeal medicine. Furthermore, K^+^ channel inhibitors like the antifungal clotrimazole, approved by the FDA for other indications, are other promising candidates [75].

#### Inhibitors of cAMP signalling

Another antidiarrhoeal strategy is to target the molecular mechanisms that regulate the activity or expression of the transport and channel proteins involved in SD. However, targeting components of the cAMP signalling cascade has the disadvantage that only a subset of them, that is GM1 receptors [76], LPA2 receptors for lysophosphatidic acid (LPA) [36, 77, 78] and, potentially, Ca^2+^‐sensing receptors (CaSRs [79]), are located on the luminal surface and are directly exposed to orally applied antidiarrhoeals. In contrast most other potential targets, including adenylyl cyclase 6 (AC6; [58]), farnesoid X receptors (FXR; [80]), somatostatin and α_2_‐adrenergic receptors [4, 40], and PDZ adaptor proteins (NHERF1‐3; [56, 68]) are located intracellularly or in the basolateral membrane and are reachable only by drugs that are effectively absorbed or administered systemically. Because none of the components of the cAMP signalling pathway is intestine‐specific, avoiding side effects of these drugs on other organs is a major challenge.

#### Inhibitors of cGMP signalling

In comparison with anticholera drugs, pharmacological inhibitors of STa‐induced SD *a priori* offer several advantages. First, STa binding to the receptor domain of GC‐C is reversible, implying that orally applied STa receptor antagonists, either peptides or small molecules, could potentially block STa‐induced secretion from the luminal side at any time during infection. Secondly, the two major molecular candidate targets, GC‐C and PKG2, are highly expressed in the intestine but are low or absent in most other tissues, explaining why resistance to STa‐induced SD is the sole phenotype of GC‐C^−/−^ mice and the major phenotype of PKG2^−/−^ mice [65, 66, 67]. Unfortunately, intensive efforts to discover STa receptor antagonists by high‐throughput screening have failed so far, but another class of GC‐C activity blockers acting intracellularly have been recently developed that inhibited STa‐provoked anion secretion in human intestinal biopsies but exhibited a ~ 100‐fold lower potency for inhibition of the atriopeptin‐target guanylyl cyclase A (GCA) and no inhibitory effect on the NO‐target soluble guanylyl cyclase (sGC) [81]. Another potential approach is the use of PKG2 blockers. A novel class of highly potent and selective PKG2 inhibitors targeting the ATP‐binding pocket was developed recently on the basis of high‐throughput screening [82]. Unfortunately their inhibitory potency in STa‐induced SD is limited for two reasons: at high concentrations, they elevate cAMP levels, conceivably through inhibition of PDE activity [82]; and in most intestinal segments, STa/cGMP induces secretion in part *via* PKA signalling which is not inhibited by the PKG2 blockers (Fig. 2) [81].

## ABC transporter inhibitor mechanisms

Inhibitors of several ABC transporters have been identified, and in some cases, studied in mechanistic and structural detail. Particularly relevant here are inhibitors of Type IV ABC systems [11], which have a TMD fold very similar to that of CFTR. Because CFTR's unique channel nature allows in depth biophysical and kinetic investigation, while other transporters are more amenable to a variety of biochemical studies, comparisons of results obtained in different systems can provide insight from different perspectives and/or highlight divergence in structure/function.

### Zosuquidar: obstructing the IF‐to‐OF transition

Binding of inhibitor zosuquidar to P‐glycoprotein (ABC‐B1) [83], and of the antidiabetic drug glibenclamide on sulfonylurea receptors (SUR1 or ABC‐C8) [18, 84] show some similarity. In both cases, the inhibitor occupies a binding site positioned in between the two structural halves and therefore acts in a manner broadly analogous to the dephosphorylated R domain on CFTR, obstructing the IF‐to‐OF transition. However, the exact binding sites are different, and while zosuquidar forms a large number of defined molecular interactions with the target transporters, the glibenclamide‐SUR1 and R domain‐CFTR interactions are looser, with the inhibitor likely adopting various conformations, suggesting differences in the details of the mechanism of action.

Cryo‐EM structures show the inhibitor zosuquidar occupying the same cavity on ABC‐B1 as transported substrates, such as taxol. But why is taxol translocated while zosuquidar interrupts the catalytic cycle? One possibility is that the zosuquidar‐ABC‐B1 interaction is too strong, restricting the protein's dynamic flexibility: unlike taxol, the inhibitor forms a larger number of contacts with the protein, fills the cavity completely and maintains a precise pose, rather than sampling a number of binding modes. Flexibility in the transporter helices might be required to allow the TM rearrangement resulting in cavity reshaping and opening of the outer gate. In addition, small structural changes at the binding cavity are allosterically transmitted and amplified (*via* TM7, TM8 and TM12) resulting in a displacement of the CHs and NBD2 away from the dimer interface (see fig. 2B–D in [83]). Curiously, despite ABC‐B1 having two canonical, catalytically active sites, using double electron resonance spectroscopy, zosuquidar is found to be unable to stabilize an asymmetric orthovanadate‐trapped (see Orthovanadate: interrupting the γ‐phosphate splitting reaction below) conformation in which one catalytic site (corresponding to canonical site 2 in asymmetric transporters) is tightly dimerized while the other is more open. How much this asymmetric conformation is stabilized correlates with how well different substrates can stimulate ABC‐B1 ATPase activity [85]. It is interesting to note that rate‐equilibrium free‐energy relationship (REFER) analysis on CFTR suggests that the highest energy conformation adopted by CFTR during the channel‐opening transition possesses an already tightly dimerized canonical site 2, while movements at site 1 are further behind [21]. It is possible that for both symmetric and asymmetric type IV transporters, adoption of an asymmetric transition‐state conformation facilitates overcoming the energetic barrier associated with the IF‐to‐OF transition. Thus, the reduced flexibility in ABC‐B1's TMDs, caused by tight binding of zosuquidar, could be precluding access to a favourable pathway in the energetic landscape linking IF and OF conformations.

### Orthovanadate: interrupting the γ‐phosphate splitting reaction

In several ABC transporters, hydrolytic activity can be inhibited by incubation with orthovanadate (V_i_), in the presence of ATP and divalent cations. The V_i_‐dependent inhibition of ABC‐B1 was the subject of biochemical studies, which elegantly demonstrated that trapping of an ADP‐Mg^2+^‐V_i_ complex in a single nucleotide binding site was sufficient to block further hydrolytic cycles at both catalytic sites [86]. The ADP‐Mg^2+^‐V_i_ complex is thought to mimic the high‐energy pentacovalent transition‐state intermediate of the phosphoryl‐transfer reaction, specifically stabilized by interactions within the active site [87, 88].

However, although V_i_ efficiently blocks the catalytic cycle of many ABC Transporters, resulting in an inhibition of pump function, V_i_ action on wild‐type CFTR channels results in an increase in overall transmembrane anion flow. Because gate closure is triggered by hydrolysis of the ATP molecule at site 2, which in turn destabilizes the NBD1/NBD2 dimer and favours a resetting of the protein to an IF conformation, V_i_ is thought to ‘lock’ channels in an open conformation (i.e., with unobstructed anion permeation pathway [14, 89, 90]), by preventing hydrolysis and thus delaying gate closure. Like Vi, pyrophosphate [91, 92], poorly hydrolysable nucleotide analogues [14] or mutations at key catalytic residues in site 2 [93] all delay gate closure by preventing/slowing hydrolysis at site 2.

While the CFTR gating cycle is clearly a nonequilibrium process [23, 94], the step corresponding to the γ‐phosphate splitting reaction is not necessarily irreversible [95], and V_i_ might reopen channels that have recently closed [96]. However, the observation that a conformational change in the permeation pathway (detected as a single‐channel conductance increase in patch‐clamp records) is prevented by the presence of V_i_, pyrophosphate, low [Mg^2+^], or catalytic site 2 mutations [97], strengthens a gating model in which hydrolysis at site 2 occurs on open channels, and is what allows the relatively fast IF resetting, and therefore hydrolytic pore closure.

### Inhibiting MsbA: obstructing IF‐to‐OF transition and NBD uncoupling

MsbA is a Type IV system, involved in translocation of lipopolysaccharide across the inner membrane in Gram negative bacteria. Inhibition of MsbA holds promise in view of novel antibiotic development. From a screen of ~ 3 million compounds, Genentech scientists identified a class of quinoline compounds capable of inhibiting MsbA and displaying potent antibacterial activity [98]. Crystal structures reveal two inhibitor molecules bound to homologous sites on an MsbA homodimer, poised in an IF conformation with relatively close NBDs. The binding pocket is lodged between TM4, TM5 and TM6 (see fig. 2 in [98]). Comparison with an OF MsbA structure [99] shows the drug‐binding pocket would be deformed in an OF conformation, suggesting that drug binding prevents the IF‐to‐OF transition. In addition, though, comparison with a drug‐free IF cryo‐EM MsbA structure [100] reveals that drug binding distorts TM4 and TM5. The distortion is transmitted allosterically along the TM pair, shifting CH2 towards the central axis of the protein (see fig. 3d,e in [98]). This movement dislocates one of the NBD/TMD ‘ball‐and‐socket’ joints: one NBD is forced out of a network of conserved interactions to avoid steric clashes with the other NBD. Thus the quinoline inhibitors could block the MsbA pumping cycle at two distinct steps: preventing the IF‐to‐OF transition but also preventing the formation of active catalytic sites at the NBD interface [98].

**Fig. 4:**
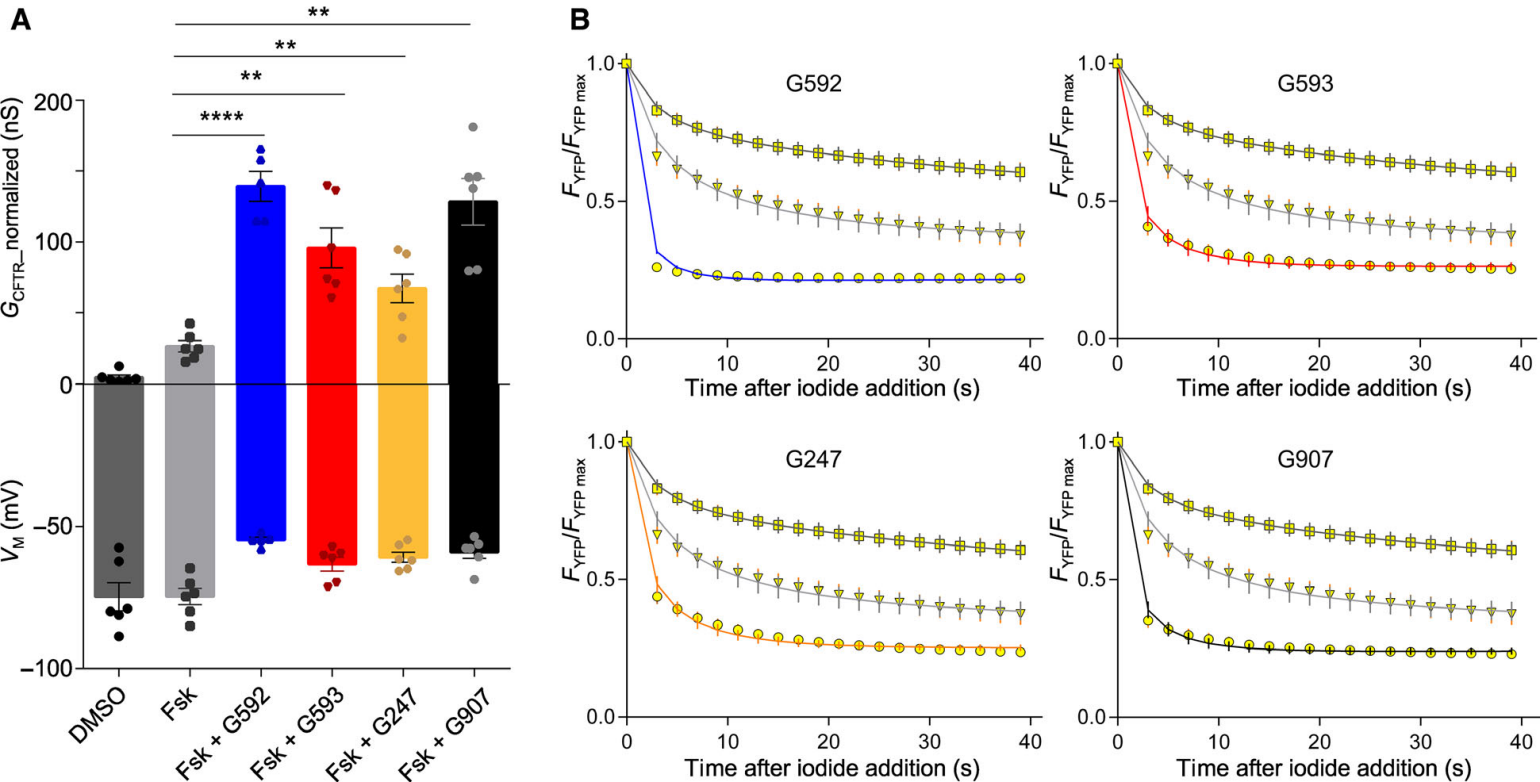
Activation of CFTR by MsbA inhibitors. Acute treatment with selective quinoline MsbA inhibitors G592, G593, G247 and G907 [98] results in an increase in cellular anion conductance. Functional analysis of wild‐type YFP‐CFTR expressed in HEK293 cells using an image‐based fluorescence microscopy assay [101, 102]. YFP‐CFTR is expressed from a pIRES2‐mCherry‐YFPCFTR plasmid, in which YFP‐CFTR and a soluble mCherry red fluorescent protein are translated from a single bicistronic mRNA. (A) Mean normalized CFTR conductance (*G*_CFTR__normalized) and cell membrane potential (*V*_M_) were estimated by fitting YFP quenching time course (see B), following extracellular addition of iodide. Before iodide addition, a 230 s pretreatment allowed CFTR activation to reach a steady state. Bars represent different pretreatment: vehicle control (DMSO, dark grey bar), 0.3 µmforskolin (light grey bar), 0.3 µmforskolin + 10 µmG592 (blue bar), 0.3 µmforskolin + 10 µmG593 (red bar), 0.3 µmforskolin + 10 µmG247 (yellow bar) or with 0.3 µmforskolin + 10 µmG907 (black bar) (*n* ≥ 5, as indicated by solid circles, each representing one measurement obtained on an independent plate). To account for possible differences in transfection efficiency between wells, CFTR conductance is normalized using the mean mCherry fluorescence measured within cells [102]. Data from wells belonging to the same 96‐well plate were paired, and paired*t*‐tests were used to determine statistical significance of comparisons (**P < 0.01; ****P < 0.0001). (B) Time course of YFP quenching following addition of extracellular iodide. Observed relative fluorescence values are shown as yellow symbols, while solid lines are fits. For each compound, graphs compare quenching time curve following pretreatment with vehicle control (squares, dark grey line), 0.3 µmforskolin alone (triangles, light grey line) and 0.3 µmforskolin + 10 µmcompound (circles, coloured line). Compound colour‐coding as in A.

Because MsbA and CFTR share a type IV system TMD fold, an image‐based fluorescence assay was used to test the effect of the quinoline compounds on anion conductance in YFP‐CFTR‐expressing HEK293 cells (Fig. 4). Time course of the anion‐sensitive YFP quenching was measured following an iodide/chloride exchange protocol [101, 102]. Unexpectedly, the compounds appear to activate CFTR, increasing cellular anion conductance and depolarizing membrane potential. Like on MsbA, the (S)‐enantiomer, G592, was more efficacious than the (R)‐enantiomer, G593 (paired *t*‐test, *n* = 5, *P* = 0.020). However, G247, which gives the highest potency on MsbA (IC_50_ = 5 nm, in which a naphthalene with a cyclopropane substituent replaces the quinoline core of G592, see fig. 1 in [98] for compound structures) appeared less efficacious on CFTR (paired *t*‐test, *n* = 5, *P* = 0.003 vs. G592). G907 (which also presents an α ‐linked cyclopropane ring) is very effective on CFTR and potent on MsbA (IC_50_ = 18 nm). Like VX‐770 [103], the quinoline compounds are very lipophilic. We cannot rule out that the micromolar concentrations tested might have given rise to nonspecific effects due to accumulation of drug in the membrane compartment. However, the significantly different effects of the equally lipophilic enantiomers G592 and G593, mirroring their differing potencies on MsbA, are more consistent with the drugs binding specifically to CFTR and increasing its activity.

One interpretation of these results is that, unlike in drug‐bound MsbA, in drug‐bound CFTR, the IF‐to‐OF transition (corresponding to channel opening in a normal CFTR gating cycle) can occur. CFTR would thus enter an NBD‐dimerized open state, but, because of the displaced NBD and altered NBD1/NBD2 interface, hydrolysis of the ATP at site 2 would be prevented, locking CFTR in an open state. However, a tight NBD dimer interface stabilizes the ground open state and transition state for the CFTR opening step [20, 22]. It is difficult to envisage how these conformations would not be destabilized by NBD uncoupling.

Alternatively, drug‐bound CFTR might be stabilized in an IF conformation similar to that observed for G907‐bound MsbA, but in which the permeation pathway is open. One of the two homologous drug‐binding sites seen on MsbA is located just extracellular to the cytosolic portal bypassing the vestigial inner gate in CFTR [32], and includes residues corresponding to M348 and R352, part of CFTR's inner vestibule [104, 105, 106]. This site is also close to the unique unwound, hinge region of TM8 (Fig. 1), a region seen to bind the approved CF potentiator drug VX‐770 [107]. The quinoline compounds might affect the dynamics of the helices surrounding the inner vestibule, favouring the yet unknown conformational changes needed to open the extracellular gate, stabilizing this open conformation and/or increasing the anion throughput. It is interesting to note that some evidence suggests that VX‐770‐bound CFTR might also have noncanonical NBD/TMD interfaces [102, 108].

## CFTR inhibitor mechanisms

**Fig. 5:**
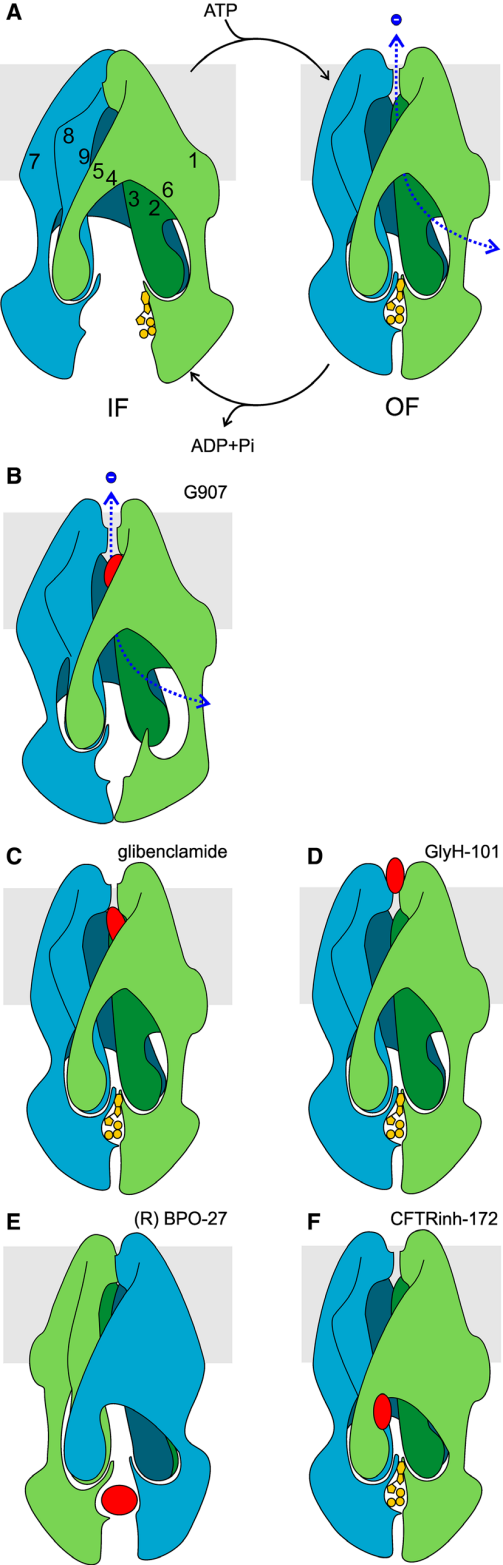
Mechanism of action of CFTR and ABC transporter inhibitors. (A) Gating of phosphorylated CFTR channels is driven by ATPase cycles. In the absence of drugs, for phosphorylated, ATP‐gated wild‐type CFTR channels, opening is coupled to formation of a tight NBD1/NBD2 dimer; while channel closing is triggered by ATP hydrolysis at site 2 (hidden in this view). In physiological conditions, degenerate site 1 is likely not as open as seen in5UAKand as depicted here [166]. (B) G907 binds between TM4,5, 6 (and/or 10, 11, 12). While it inhibits MsbA, it increases anion conductance of CFTR‐expressing cells. Drug binding to CFTR is here hypothesized to alter position of coupling helices, bringing them closer together, and forcing a dislocation of ball‐and‐socket joint with NBD1. Conformational changes at the extracellular end of the inner vestibule result in an opening of the permeation pathway. (C) Glibenclamide is an open‐pore channel blocker, whose binding site is within the membrane electric field. It accesses its binding site from the cytosol, and apparent affinity is increased by hyperpolarization. (D) At low micromolar concentrations, GlyH‐101 is a nonabsorbable open‐pore channel blocker, which acts from the luminal side of the membrane. Its binding is favoured at depolarized membrane potential. (E) BPO‐27 competes with ATP for binding at canonical site 2, preventing the IF‐to‐OF transition, and therefore channel opening. (F) Binding of CFTR_inh_‐172 triggers a conformational change that leads to channel closure (without requiring dissociation of NBDs). Here, this conformational change is shown to close the cytosolic portal between TM4 and TM6.

Considering its crucial role in CT‐, LT‐, and STa‐induced Cl^−^ secretion, CFTR is a major target for the development of antisecretory drugs. Multiple small‐molecule CFTR inhibitors have been identified [6, 7, 8, 9], and mechanism of action for a number of these have been investigated (Fig. 5).

### Glibenclamide: inner vestibule pore block

Glibenclamide, an antidiabetic drug targeting SUR1 (ABC‐C8) in pancreatic β cells, has been shown to inhibit CFTR when present on the cytosolic face of the membrane. Patch‐clamp records and alterations of gating kinetics are consistent with an open‐pore block [109, 110]: binding of the drug within the permeation pathway in open channel conformations, obstructs anion flow. The anionic form is the active blocker, and hyperpolarizing membrane potential favours block, suggesting a blocker binding site located in the inner vestibule of the pore, within the electric field of the membrane [110]. Similar voltage‐dependence, sensitivity to mutation and mutually competitive interaction [111] suggest that a number of other organic anions acting as blockers are subjected to similar electrostatic interactions with positively charged residues, including K95 [112], lining the relatively spacious inner vestibule. In addition, permeant anions, still carrying their hydration shells, also likely compete for this dynamic binding site [113] along the permeation pathway, explaining the influence of the Cl^−^ gradient on glibenclamide block [110].

While we do not have experimental structures confirming these functional inferences on CFTR, the binding of glibenclamide to SUR1 has been studied by cryo‐EM. Density corresponding to glibenclamide is seen in the central cavity between the transmembrane domains of the core of SUR1, which adopts an IF conformation. Bound to SUR1, glibenclamide prevents Mg^2+^‐nucleotide stimulation of ATP‐sensitive K^+^ channels (K_ATP_) channels. It has been suggested that bound glibenclamide might hinder NBD dimerization and the IF‐to‐OF transition in SUR1 [114]. This hypothesis is consistent with the glibenclamide‐occupied cavity shrinking in an NBD‐dimerized, Mg^2+^ nucleotide‐bound conformation [18].

However, the open‐pore block characteristics of glibenclamide inhibition of CFTR suggest that the drug binds to the NBD‐dimerized, OF open channel [20] conformation. The two ABC‐C subfamily proteins have a similar overall core fold in TMD1 and TM10/12 (CFTR) and 15/17 (SUR1), and several glibenclamide‐contacting SUR1 residues (Y377, R1246, W1297 [115]) are conserved in CFTR (I142, R1097, W1145, respectively). The discovery of density corresponding to the N terminus of the Kir6.2 subunit in this region of the SUR1 central cavity [115], alongside glibenclamide, raises a plausible scenario: as in ABC‐B1, the TMs surrounding the glibenclamide/substrate binding cavity might need to undergo rearrangements during the IF‐to‐OF transition, and the simultaneous presence of the Kir6.2 N terminus and the drug might be what is preventing these in SUR1. In CFTR, lacking the inserted N‐terminal peptide, the IF‐to‐OF transition can occur, giving rise to the pore‐blocking glibenclamide binding site, at a more extracellular position in CFTR than in the glibenclamide‐(SUR1‐Kir6.2)_4_ complex. The TM rearrangement could also allow a reorientation of the elongated glibenclamide molecule, so as to position the charged sulfonylurea end of the drug close to K95 [112] and several positively charged TM6 residues [116] lining the intracellular vestibule (also cf. [117]).

### Glycine hydrazides: extracellular pore block

The development of high‐throughput assays to test CFTR function in the Verkman laboratory [118] opened the way to screening of large compound libraries for the identification and development of high‐potency CFTR inhibitors. Glycine hydrazides were among the first that were identified and a first compound, GlyH‐101 with low micromolar potency, was developed. Again, kinetic analysis suggested that the inhibitor bound to the open channel, causing pore block, and the voltage‐dependence of inhibition suggested a binding site partly within the membrane electric field. However inhibition occurred following extracellular application and apparent potency was increased by depolarization of membrane potential [119]. Slowing down of rates of reaction of thiol‐reactive compounds with F337C‐ and T338C‐CFTR, following exposure to GlyH‐101 [120], confirmed binding to a site close to residues F337 and T338, positioned at the extracellular end of the narrowest region of the permeation pathway [121].

All these results are consistent with glycine hydrazides binding in the outer mouth of the pore and causing open‐pore blockage. While a target site positioned on the luminal face of enterocytes has some pharmacokinetic advantages, bypassing the requirement of systemic absorption, mathematical modelling suggests that only compounds possessing extremely high potency could achieve the high levels of inhibition required in the presence of the intense convective washout fluxes typical of SD pathology [122]. A number of macromolecular conjugates of glycine hydrazides have been developed (e.g. [123]). These, by adding a moiety which binds to the glycocalyx of enterocytes, resist intestinal washout. However, macromolecular conjugate drugs are not cheap to produce nor stable without refrigeration, effectively limiting the practical utility of these compounds for treatment on the ground. A further potential obstacle in the therapeutic use of GlyH‐101 is its cytotoxicity observed at suprapharmacological concentrations [124] (seen also for CFTR_inh_‐172).

### Quinoxalinediones: competition at ATP‐binding sites

Pursuing the quest for extremely high‐potency compounds, the Verkman laboratory recently identified another class of CFTR‐inhibiting compounds, the PPQ/BPO compounds. Structure activity relationship studies and chemical optimization led to the benzopyrimido‐pyrrolooxazinedione BPO‐27 molecule with an IC_50_ below 10 nm.

The (R) enantiomer was found to be the active compound [125], and patch‐clamp studies confirmed an IC_50_ below 5 nm for this stereoisomer. Application of the inhibitor to the cytosolic face of the patch caused a lengthening of the interburst closed dwell‐times. In addition, dose‐response curves of currents obtained with increasing concentrations of ATP were shifted to higher [ATP] by the presence of (R)‐BPO‐27. The results support a mechanism of action in which (R) BPO‐27 competes with ATP at the canonical ATP‐binding site, site 2, but drug binding fails to trigger conformational changes resulting in opening of the gate [126]. Docking and molecular dynamics studies confirmed the presence of a favourable binding pocket at the interface between the P‐loop of NBD2 and the signature sequence of NBD1 in an IF CFTR homology model [33].

A study using mice intestinal loops and intestinal epithelia demonstrated the efficacy of BPO‐27 in preventing CT‐ and STa‐ induced luminal accumulation of fluid. No obvious toxicity was noticed in mice, at concentrations giving adequate CFTR inhibition [127]. However, no careful investigation of selectivity was carried out.

The consensus ATP‐binding site is extremely conserved in ABC transporters [11]. Because the residues predicted to interact with BPO‐27 are part of conserved motifs (P‐loop, A‐loop, signature sequence) selectivity for CFTR over other members of the superfamily needs to be verified. For instance, acute toxicity might result due to BPO‐27 competing with Mg^2+^‐ATP regulation of K_ATP_ channels (8/10 of the interacting residues are identical in CFTR, ABC‐C8, and ABC‐C9) or BPO‐27 interference with the immune response due to inhibition of TAP1/2 (binding site residue identity: 6/10; conservation: 8/10).

### Thiazolidinone: favouring closing of the inner gate?

The first micromolar potency, CFTR‐selective (not affecting ABC‐B1 or K_ATP_ activity), inhibitor discovered through high‐throughput screening was the thiazolidinone CFTR_inh_‐172 [128], the mechanism of action of which has been studied in depth.

Initial electrophysiological studies showed no effect of the drug on single‐channel conductance, nor on open dwell‐times. In addition, inhibition is voltage‐independent – all characteristics suggesting a mechanism distinct from open‐pore block. Instead, inhibition reflects a reduction in open probability, and in particular a prolongation of interburst closed dwell‐times. However, unlike for BPO‐27, inhibition by CFTR_inh_‐172 is not affected by [ATP] [129].

More in‐depth studies revealed that CFTR_inh_‐172 not only prolongs closed dwell‐times but the compound also affects open dwell‐times, with increasing concentrations of the drug causing a progressive shortening to a minimum. Inventive experiments demonstrated that binding of the inhibitor could occur on both open and closed channels, but apparent affinity increased in conditions known to stabilize the NBD‐dimerized open state (catalytic site 2 mutations, pyrophosphate, P‐ATP). This suggested a tighter binding of the inhibitor to OF conformations. Binding of the inhibitor did not prevent ATP control of the extracellular gate, mediated by NBD dimerization. For instance, ATP could remain trapped in inhibitor‐closed channels, revealed by their reopening, upon inhibitor washout, without further addition of ATP; Inhibitor‐bound closed channels could open, upon ATP addition, but inhibitor presence was revealed by the shorter open dwell‐times. Thus, although CFTR_inh_‐172 seemed to close a gate distinct from the ATP‐controlled gate, the dynamics of the two gates were found to be coupled. These considerations led Kopeikin and colleagues to suggest that CFTR_inh_‐172 might be favouring the closing of the vestigial ‘inner gate’, homologous to structures preventing substrate from having access to the cytosol in OF conformations of CFTR's pump relatives [130].

Mutations that remove the positive charge at R347 (R347A, R347D) cause a dramatic decrease in CFTR_inh_‐172 potency, even when an open‐pore stabilizing salt bridge [131] is maintained (R347D/D924R) [132]. The R347D mutation also reduces the potency of a modified compound in which the negative charge is removed, arguing against the charge of R347 interacting directly with the carboxyphenyl group of the bound inhibitor [132]. An allosteric effect of the mutation seems more likely. Interestingly, the R347D mutation has been shown to reduce ATPase activity [133]. The mechanism by which structural changes introduced by the mutation (near the extracellular gate) are transmitted to the catalytic site (at the NBD interface) is unknown. But it is plausible that R347D might allosterically interfere with NBD dimerization. If so, the effects of the R347D mutation on CFTR_inh_‐172 potency [132] would be consistent with the observed correlation between propensity to adopt OF, NBD dimerized, conformations and apparent affinity [130]. Whether CFTR_inh_‐172 binding to CFTR triggers conformational changes that close off the lateral portal between TM4 and TM6 thus remains a question worth further investigation.

## Future development of therapies for treatment of SD

### Challenges

Considering the predominant incidence of diarrhoeal diseases in developing countries, implying a low profit potential for pharmaceutical companies, funding of the development costs and costs of field trials for novel antisecretory drug candidates remains a major challenge. Global cooperation between developed and developing countries and philanthropic donations are needed to overcome these hurdles. Repurposing drugs that are already approved for other diseases or the use of natural products could be a cost‐effective alternative strategy. In this respect, traditional medicine such as a commonly used Thai herbal remedy [134], the diterpenoid Oridonin [135], or Crofelemer, a proanthocyanidin oligomer isolated from the South American *Croton lechleri* plant [136], all acting in part as CFTR inhibitors, are attractive and inexpensive candidates.

When and where SDs are most lethal, good hygiene conditions and efficient medical infrastructure are not always available. Programmes aimed at developing an effective CFTR‐targeting pharmacological treatment, to complement existing therapies, will need to take this reality into account. In order for stocks to be available and replenished regularly in remote, rural communities worldwide, drugs would need to have a low production cost, affordable to the health systems of developing countries. The active small‐molecule would also need to be stable, requiring little or no refrigeration or protection from excessive humidity. Finally, oral bioavailability is also an advantage, not requiring skilled medical staff, nor high levels of cleanliness and hygiene for safe administration. Given these requirements, pharmacokinetic optimization will inevitably require considerable resources and will play a significant part in the potential success of therapy on the ground.

**Fig. 6:**
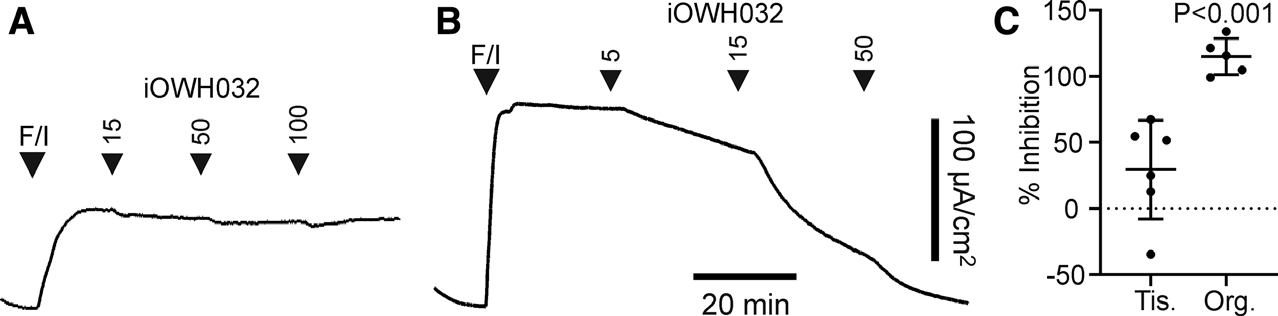
Different potency of GlyH‐101 analogue iOWH032 in human rectal biopsies as compared to 2D rectal colonoids originating from the same individual. (A) Rectal biopsy specimens obtained from healthy individuals were mounted in Ussing chambers and short‐circuit currents (*I*_sc_), representing CFTR‐mediated anion secretion, were assessed as described in detail elsewhere [81, 82]. iOWH032, a GlyH‐101 analogue [40], was added only to the luminal bath; the cAMP agonist forskolin (F; 10 µm) and 3‐isobutyl‐1‐methylxanthine (I; 100 µm) were added to both the luminal and basolateral bath. (B) Undifferentiated colonoid monolayers derived from the rectal biopsies shown in panel A were grown on Transwell filters and subsequently mounted in Ussing chambers.*I*_sc_measurements were performed as described for panel A. (C) iOWH‐032 mediated inhibition of the cAMP‐dependent*I*_sc_response in colon tissue (Tis.) and organoids (Org.), as assessed at the maximal iOWH032 concentration tested in panels A and B. Horizontal bars depict mean ± SD of 6 (Tis.) or 5 (Org.) experiments. Data were statistically evaluated by ANOVA. The improved efficacy of iOWH032 in colonoid monolayers as compared to the rectal biopsies may have multiple causes: (a) the flat structure of the monolayer, preventing convective washout of the inhibitor as occurs in intestinal crypts [122]; (b) the lack of goblet cells and therefore of a mucus barrier in undifferentiated intestinal organoids [167]; (c) CFTR is rate‐limiting for the forskolin/cAMP‐induced anion secretory current in colonoids but not in rectal biopsies from healthy individuals [38].

Unfortunately, many of the CFTR inhibitors so far identified, upon oral administration, poorly penetrate into the deep intestinal crypts, the major source of CFTR‐mediated fluid secretion, because of convective washout with secreted fluid and abundant mucus [122]. Furthermore, because in the intestine from healthy individuals, in contrast to CF patients, CFTR is not rate‐limiting for transepithelial Cl^−^ and fluid secretion, inhibition of CFTR activity needs to reach high levels (probably > 80%) before the inhibitor starts to be effective [38]. Both considerations together most plausibly explain why the luminal addition of such small‐molecule CFTR blockers does not effectively block cAMP‐induced Cl^−^ secretory currents in human rectal biopsies but potently inhibits CFTR‐mediated secretory Cl^−^ currents in monolayers of intestinal organoids generated from these biopsies (Fig. 6).

Approaches to improve their efficacy may include the combined use of mucolytics, or the encapsulation of CFTR inhibitors inside acid‐resistant nanoparticles. Ideally, to prevent the systemic bioavailability of the drugs and to restrict their action to the intestine, the nanoparticles should be designed in such a way that they become entrapped and degraded within lysosomes rather than being exocytosed at the basolateral membrane of the enterocyte [137]. Mucus‐coated human 2D intestinal organoids (see below) are excellent novel models to test such approaches.

Chronic administration of CFTR inhibitors might raise concerns about toxicity (e.g. turning SD into CF [138]), However, short‐term treatment, preferably as an adjuvant therapy to ORS, is expected to be sufficient to prevent extreme fluid loss and to be life‐saving in this self‐limiting disease. Moreover, testing CFTR inhibitors in mouse models of SD [139] and for the inhibition of cyst growth in polycystic kidney disease [140], did not reveal evidence of serious side effects. However, this does not preclude the occurrence of CF‐like pathology in humans, considering the more severe lung and pancreatic phenotype in human CF patients. Human testing will be important, especially given the extreme susceptibility of CF patients to infection and inflammation.

### Opportunities

Despite the considerable limitations of current drugs targeting CFTR and of therapies for SD in general, recent advances in biotechnology and in the use of computational tools could be instrumental in overcoming some challenges and identifying lead compounds for new effective small‐molecule therapies.

Human enteroids present a novel tool to study human intestinal ion transport physiology and pathophysiology, as well as permitting novel and personalized screens [141, 142]. These ‘mini‐gut’ structures can be generated from the stem cells in intestinal biopsies and grown in virtually unlimited amounts, either 3‐dimensionally (3D) in Matrigel droplets, or 2‐dimensionally (2D) on matrix‐coated filters or micro‐engineered gut‐on‐a‐chip devices [143, 144]. The latter technique can be used also to mimic peristalsis and fluid flow. Whereas enteroids and colonoids remain reductionist models, that is are missing the complexity of the native tissue, this limitation can be overcome by co‐culturing the organoids with other cell types such as microbes, immune cells, neural crest cells and mesenchymal cells [144, 145, 146]. Although ‘basal out’ 3D organoids were the first models to demonstrate, on the basis of CT‐ or forskolin/cAMP‐induced fluid secretion (FIS) assays, a high functional expression of CFTR and its inhibition by CFTR inhibitors [142], the poor accessibility of their luminal compartment to enterotoxins (CT, STa) and to CFTR‐ or CT‐specific inhibitors makes them less suitable for antidiarrhoeal drug screening. In contrast, 2D monolayers of enteroids and colonoids are freely accessible at both the basolateral (serosal) side and the apical (luminal) side, therefore bypassing the need for microinjection of test compounds or microbes. Moreover, unlike 3D organoids they allow traditional ion transport studies, including electrical measurements of CFTR‐mediated Cl^−^ secretory currents in Ussing chambers and NHE3 activity measurements by multi‐photon microscopy [147, 148]. Human intestinal organoids offer many advantages compared with the cancer cell lines traditionally used in intestinal ion transport studies. First, they are unique in their capacity to allow personalized theratyping of drugs that could be applied in ion transport diseases such as cystic fibrosis and diarrhoeal disease [149, 150]. Second, they can be used to study segmental differences and spatial differences along the crypt‐villus axis at both a macroscopic and microscopic level [151]. Third, goblet cell‐enriched organoid monolayers are capable of building up a mucus layer at their luminal side which mimics the barrier function of native epithelium [152, 153]. This allows the assessment of the impact of the mucus barrier on the efficacy of luminally added compounds, including CFTR inhibitors. Moreover, approaches that aim to facilitate transport of antidiarrhoeal agents across the mucus layer, such as alginates or nanoparticles, can be evaluated [154, 155].

The past decades have seen a major increase in the number of full‐length ABC transporters for which we have atomic‐level models of 3D structure. Boosted by this detailed information, a large number of functional studies have been carried out, developing, refining and testing hypotheses on how these proteins function. In addition, for CFTR, the identification and development of modulator drugs for treatment of CF has also resulted in new tools, improving our ability to investigate structure/function questions. We now have a deeper understanding of the protein structure and of the dynamics underlying channel function [1]. Guided by this knowledge, computational simulations and *in silico* screening algorithms will soon be used much more effectively to identify and develop drugs targeting CFTR.

Based on the molecular mechanisms by which small molecules can affect CFTR (Fig. 5), which of the conformational changes underlying the gating cycle should we attempt to target? Intervention at what steps would be most suited for reversibly reducing anion flow mediated by CFTR?

Preventing or slowing down the IF‐to‐OF transition coupled to the opening of the CFTR pore might be an achievable target. For CFTR this is the slowest step of the gating cycle (e.g. [23]), probably reflecting the large conformational change required, thus small changes in this microscopic rate could achieve large effects on open probability. Several small molecules (including zosuquidar, glibenclamide and the quinoline MsbA inhibitors) are thought to act at this step on other ABC systems, by binding to pockets formed in between the transmembrane helices. CFTR's unique TMD2 structure, including an unwound TM8 might provide an opportunity for selectivity. However, for the moment, rational design of such small molecules is not possible, because we do not know the final conformational change underlying gate opening, nor the detailed structure of the extracellular end of the CFTR permeation pathway. This gap in our knowledge might be filled soon as structural biologists prepare single particles for cryo‐EM analysis using lipid nanodiscs (rather than detergent solubilization) to embed these proteins in an environment closer to a lipid bilayer membrane. In the meanwhile, an alternative strategy could be to exploit ligand‐based virtual screening tools [156, 157], using existing ligands as starting points for a focused exploration of chemical space. Chemical analogues identified *in silico* could be screened for effects with 2D organoids and other high‐throughput biological assays [102, 158, 159]. CFTR ligands thought to act at this step, albeit normally speeding up channel opening (e.g. the quinoline compounds [98], 5‐nitro‐2‐(3‐phenylpropylamino)benzoate [160, 161] or VX‐770 [162, 163, 164]), could also provide useful focus points, aiming at identifying analogues acting as reverse agonist. Finally, the putative mechanism of action of CFTR_inh_‐172 also suggests a conformational step, modulation of which is worth exploring. The opening between TM4 and TM6, corresponding to CFTR's lateral portal, is unique to the channel CFTR, and conservation in this region – even among asymmetric ABC transporters [15] is low. Small molecules, binding at this site and triggering portal obstruction, could be designed to reach a very high affinity, with minimal risk of simultaneously increasing off‐target affinity.

## Conclusions and perspectives

Among the many antidiarrhoeal therapies discussed briefly in this review, inhibition of CFTR by orally bioavailable compounds could potentially be highly effective against SD caused by noninvasive pathogens such as *V. cholerae* and ETEC strains. If readily available as emergency treatment, it could prevent death by dehydration in particularly vulnerable individuals, such as children, and slow the spread of infection. However, the proper targeting of these inhibitors to CFTR in the intestinal crypts, hampered by their convective washout with secreted fluid and abundant mucus, remains a major challenge. Another intrinsic limitation of therapies based on CFTR inhibitors is their inability to reactivate NHE3‐mediated NaCl and fluid absorption, in contrast to inhibitors of cAMP or cGMP signalling. However, this constraint can be overcome by combining CFTR inhibitor therapy with ORS, thereby exploiting Na^+^‐coupled glucose and fluid absorption, which is not inhibited by the enterotoxins.

Finally, aside their potential therapeutic applications, CFTR inhibitors can also be used as tools to elucidate CFTR gating dynamics and ABC transporter mechanisms. As highlighted in this review, some compounds can alter the function of a number of ABC systems and comparing effects can lead to better mechanistic understanding, which in turn could guide improvement of therapies. Glibenclamide prevents the IF‐to‐OF transition in SUR1, but not in CFTR. Further investigating whether this reflects CFTR's unique structure and protein dynamics at the extracellular end of the TMDs (extracellular gate) or specific SUR1‐Kir6 interactions could have an impact on therapies targeting both proteins. The MsbA inhibitors appear to activate CFTR. As such, development of the inhibitors for antibacterial therapy will need to take into account any off‐target effects on CFTR. On the other hand, further investigation of their action on CFTR could help clarify the still poorly understood opening of the extracellular gate.
